# Socio-economic status and types of childhood injury in Alberta: a population based study

**DOI:** 10.1186/1471-2431-6-30

**Published:** 2006-11-09

**Authors:** Susan J Gilbride, Cameron Wild, Douglas R Wilson, Lawrence W Svenson, Donald W Spady

**Affiliations:** 1 University of Alberta, Edmonton, Canada; 2 Department of Public Health Sciences and Centre for Health Promotion Studies, University of Alberta, Edmonton, Canada; 3 Department of Public Health Sciences, University of Alberta, Edmonton, Canada; 4 Health Surveillance Branch, Alberta Health and Wellness, and Department of Public Health Sciences, University of Alberta, Edmonton, Canada; 5 Department of Pediatrics and Public Health Sciences, University of Alberta, Edmonton, Canada

## Abstract

**Background:**

Childhood injury is the leading cause of mortality, morbidity and permanent disability in children in the developed world. This research examines relationships between socio-economic status (SES), demographics, and types of childhood injury in the province of Alberta, Canada.

**Methods:**

Secondary analysis was performed using administrative health care data provided by Alberta Health and Wellness on all children, aged 0 to 17 years, who had injuries treated by a physician, either in a physician's office, outpatient department, emergency room and/or as a hospital inpatient, between April 1^st^. 1995 to March 31^st^. 1996. Thirteen types of childhood injury were assessed with respect to age, gender and urban/rural location using ICD9 codes, and were related to SES as determined by an individual level SES indicator, the payment status of the Alberta provincial health insurance plan. The relationships between gender, SES, rural/urban status and injury type were determined using logistic regression.

**Results:**

Twenty-four percent of Alberta children had an injury treated by physician during the one year period. Peak injury rates occurred about ages 2 and 13–17 years. All injury types except poisoning were more common in males. Injuries were more frequent in urban Alberta and in urban children with lower SES (receiving health care premium assistance). Among the four most common types of injury (78.6% of the total), superficial wounds and open wounds were more common among children with lower SES, while fractures and dislocations/sprains/strains were more common among children receiving no premium assistance.

**Conclusion:**

These results show that childhood injury in Alberta is a major health concern especially among males, children living in urban centres, and those living on welfare or have Treaty status. Most types of injury were more frequent in children of lower SES. Analysis of the three types of the healthcare premium subsidy allowed a more comprehensive picture of childhood injury with children whose families are on welfare and those of Treaty status presenting more frequently for an injury-related physician's consultation than other children. This report also demonstrates that administrative health care data can be usefully employed to describe injury patterns in children.

## Background

Childhood injury is a major public health concern throughout developed countries and is the leading cause of mortality, morbidity and permanent disability in children Injury-related mortality rates among Canadian children and youth are second highest out of eight industrialised countries, and only the United States has a higher rate of injury related deaths among children [1]. It is apparent that some children are more at risk for injury than others, especially those from lower socio-economic families [2,3]. The causes of this additional vulnerability include a complex array of behavioural, social, and environmental factors.

The first goal of the present study was to examine injury-related outcomes beyond mortality. While many studies have examined injury mortality among children [4-7] mortality is only the tip of the injury pyramid. Other, more common, outcomes of injuries include major trauma with prolonged hospitalisation and rehabilitation along with use of emergency room and primary healthcare services. For example, in 1995, 1,397 Canadian children and youth (0–19 years of age) died as a result of injuries, while fully 47,228 were hospitalised for injuries [2]. Childhood injuries treated at home and in schools are even more common and define the base of the injury pyramid [8]. We believe that a comprehensive and complete description of childhood injury has yet to be definitively established, because most research on this topic emphasises mortality rather than these other, more common outcomes. Thus, the present study focused on all children who presented for a physician consultation, either in a physician's office, outpatient department, emergency room and/or as a hospital inpatient, in a year.

Another limitation of previous work in this area is that most studies of childhood injury have examined either overall rates of injury [5], specific causes of injury [7], or have narrowly focused on one particular type of injury [9]. One exception was Brownell et al.'s study [10], which divided childhood injury into 14 categories based on external cause of injury. We believe that a comprehensive and complete description of childhood injury would also be facilitated by examining a variety of different injury types. Thus, the second goal of the present study was to examine different types of child injury in Alberta by analysing International Classification of Diseases, 9^th^. Revision (ICD-9), codes [11] in relation to physician consultations.

The third goal of the study was to examine relationships between socio-economic status (SES) and childhood injuries. There is growing evidence that lower SES is associated with injury mortality among children [4-7]. Socio-economic status refers to one's social and economic position in society, and is often expressed using ordinal scales (e.g., income, occupation or educational level obtained). However, the process of choosing the most appropriate SES indicator is difficult and consequently, the variables used to define SES vary from study to study. Unfortunately, few countries have adequate data relating childhood injury to SES [3] and, to date, there have been no studies addressing specifically the relationship of SES and childhood injury in the province of Alberta. While most studies operationalize SES using an ecologic measurement, i.e., census data [12,13], a recent study [14] suggests that this approach tends to reduce differences when compared with individual measures of SES. In the present study, our dataset tracked all children's visits to a physician and itemised the payment status of the Alberta provincial health insurance plan which can be used as a proxy indicator for individual level SES. Thus, the present study used payment status as a proxy indicator for individual-level SES.

When examining environmental factors associated with childhood injury the location of the home may be an important factor. Brownell et al. [10]reported that children living in rural Manitoba had significantly higher rates of hospitalisation for injuries than those living in the urban Canadian city of Winnipeg. The final goal of the present study was to determine whether there were similar urban/rural differences in the province of Alberta.

### Research Questions

The administrative health database used in the present study allowed us to analyse all physician consultations and types of injury among children in a 12-month service period. We performed secondary analysis of this database to address four research questions: (1) Are children of lower SES more likely to present for a physician consultation with an injury compared to children with higher SES? (2) What is the relationship between different types of childhood injury and the child's SES? (3) What is the relationship between the different types of childhood injury and different types of healthcare premium subsidy? and (4) Are there differences in the number of childhood injuries between those living in rural and urban Alberta?

## Methods

Alberta, Canada is a culturally diverse, prairie province with a population of about 3 million. About 75% of its population live in urban centres. The province has an abundance of natural resources and has one of the strongest economies in North America. The province of Alberta maintains a publicly-funded universally available healthcare system for all residents of the province. All adults in the province of Alberta and their children are required to register with the Alberta Health Care Insurance Plan (AHCIP). While members of the military, Royal Canadian Mounted Police and federal inmates are excluded from the AHCIP, their dependents are required to register. It is estimated that >99% of eligible children are registered with Alberta Health and Wellness (AHW) [15]. For this study, secondary analysis was performed using data from AHW on all children who had injuries treated by a physician during a one-year period. By examining diagnoses recorded in the Provincial health administrative database, this cross sectional study identified differences in age, gender, rural/urban residence and SES for children who sought a physician's consultation for an injury from those that visited a physician for another reason. These data also allowed for an examination of these variables in relation to different types of injuries.

### Participants

The study population consisted of all children, age 0 to 17 years, registered with the AHCIP, who were seen by a physician during a fiscal year (1995–96) either in a physician's office, outpatient department, emergency and/or as a hospital inpatient.

### Procedure

This study received ethical approval from the Health Research Ethics Board at the University of Alberta. Two administrative data sets were received from AHW: service data and registration data. The service data contained the following elements: service start date (April 1^st ^1995 through March 31^st ^1996, inclusive), primary diagnosis, service location, and a unique patient identifier. The registration data contained information about the gender of the child, integer age as of March 31^st ^1996, postal code, healthcare insurance premium status, and a unique identifier. These two data sets were merged using the unique identifier as the common denominator.

### Measures

#### Place of residence

To determine the child's place of residence, postal codes were examined. A postal code with a 0 as the second character denotes a rural address (classified as less than 4000 points of call); the remainder were classified as urban.

#### Socio-economic status

A measure of SES was derived from the AHCIP premium subsidy eligibility information. Alberta charges a healthcare insurance premium and provides reductions of this premium for low income Albertans. Non-payment of the insurance premium does not impact a person's ability to access necessary care. Three low SES variables were defined. First, individuals or families who have a low income qualify for partial or total healthcare premium subsidies. Second, First Nation's children who have Treaty status (i.e. people registered under the Indian Act of Canada) have their healthcare premiums paid for by the Federal government regardless of their income, but were included in the low SES group as they live in an environment where poverty is common. Third, families living on welfare, have premiums waived and are supported by social services (Alberta Department of Human Resources and Employment). The qualifying levels for the premium subsidy in the benefit period July 1^st ^1995 until June 30^th ^1996 were based on the 1994 adjusted taxable balance; a family with a taxable income of $7,500 or less received a full subsidy, a partial subsidy was granted if the income was between $7,501 and $12,620 [16]. The three categories of families who received a subsidy (where the premium was partly or totally paid), who were of Treaty status, or were supported by social services (families on welfare), were grouped together as receiving a subsidy and were classified as low SES (n = 170,942), and those that had received no financial assistance with healthcare premiums were classified as receiving no subsidy and were classified as the normal SES level (n = 578,982).

#### Injury

Injury episodes were classified using the World Health Organization International Classification of Diseases, 9^th^. Revision, codes 800 to 999 [11]. These injuries were then collapsed into 13 revised codes to define patterns of injury and allow for a simpler description of injury types [17].

### Analysis Plan

Secondary analysis of the data was performed using the Statistical Package for Social Sciences (SPSS) 10 and Stata. Three types of analyses were conducted: (1) rates of injury, calculated using the number of events for a specific injury type as the numerator and the number of children potentially exposed to injury as the denominator, (2) the relative frequency of each type of injury, calculated as a proportion of total injuries, and (3) logistic regression to determine the relationship between injury and SES and rural/urban status and SES after adjustment for age and gender.

## Results

### Demographic Characteristics

Table 1 outlines demographic characteristics of children who were registered with Alberta Health and Wellness during the 1995–96 fiscal year. A total of 182,759 children were seen by a physician for an injury one or more times during the year, a 24% subset of 749,924 registered children.

**Table 1:** Demographic characteristics of children aged 0 to 17 years registered with Alberta Health and Wellness during the 1995–96 fiscal year.

	**Registered Population**	
**Variable**	**N**	**%**

Total number of children	749924	100.0%	
Gender of the child		
M	365509	48.7%	
F	384415	51.3%
Age of the child		
Less than one year	38478	5.1%
1 to 4 years old	163184	21.8%
5 to 9 years old	215835	28.8%
10 to 14 years old	213959	28.5%
15 to 17 years old	118468	15.8%
Place of residence		
Urban	549261	73.2%
Rural	200663	26.8%
Healthcare premium		
No subsidy	578982	77.2%
Any subsidy	170932	22.8%
Partial or total	94549	12.6%
Social services	34595	4.6%
Treaty status	41798	5.6%

### Overall Injuries

Overall injury rates per 1000 children registered with AHW were calculated to compare injury in relation to age groups, gender, urban/rural residence, and SES (Table 2). Injuries were more frequent among male children. In relation to age, injury rates were disproportionately lower in children less than 1 year, while disproportionately high in ages 15–17. Injuries were also more frequent in urban settings. There was little difference in injury rates between children receiving a premium subsidy (246.26/1000) and those not receiving a subsidy (242.95/1000), but injuries were less frequent in the sub-group receiving a partial or total premium subsidy, and significantly more frequent in the sub-groups receiving social services or with Treaty status (Table 2).

**Table 2:** Overall injury rates per 1000 children.

	**Age <1**	**1–4 y**	**5–9 y**	**10–14 y**	**15–17 y**	**0–17 y**
Overall	57.85	238.30	201.62	278.51	325.34	243.70
Gender						
Males	62.66	270.40	232.27	317.30	386.08	280.28
Females	53.23	207.75	172.41	241.62	268.05	208.93
Residence						
Urban	58.18	244.17	204.86	285.60	325.38	247.49
Rural	56.87	221.49	192.79	259.81	325.22	233.35
SES						
No subsidy	55.41	233.13	197.44	278.71	326.62	242.95
Any subsidy	64.49	253.01	215.44	277.73	320.16	246.26
Partial or total	59.30	235.62	198.69	254.31	301.12	228.01
Treaty status	66.24	267.79	231.25	300.87	330.47	261.52
Social services	77.27	284.02	240.93	313.46	359.20	277.73

#### Age, gender and childhood injury

**Figure 1:**
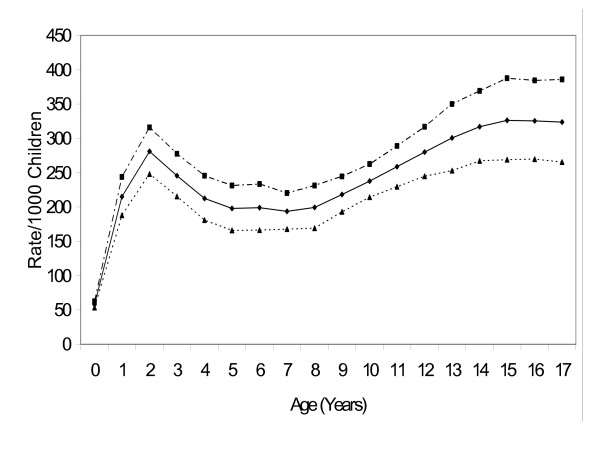
**Rates of Injury in relation to Gender**. ■ indicates males, ▲ indicates females, ◆ indicates all children.

Table 2 indicates that injury rates were higher for males of all age groups. Figure 1 illustrates this finding and shows the differences in injury rates during the childhood years with the highest levels at ages 13–17 years and also higher rates at 2 years of age.

#### Age, residence and childhood injury

Children living in urban Alberta were treated for injury more frequently than rural children, particularly between 1 and 14 years of age (Table 2).

#### Age, SES and childhood injury

Children from low SES families, up to age 9 years, experienced higher rates of injury than those from a higher SES (Table 2). In all age groups children living on welfare (social services) or of Treaty status had substantially higher rates of treated injuries compared with all other children.

### Types of Injury

The frequency and rates of each of the 13 types of injury are shown are in Table 3, which also presents the ICD codes used to define individual injury types. The great majority of records only contained one ICD9, and for the purpose of this study, if there were multiple codes only the first code associated with the injury was counted. The four most common types of injury listed in Table 3 accounted for 77.9% of all injuries.

**Table 3:** Types of injury.

**Type of Injury**	**ICD 9 code**	**Frequency**	**Percentage of total injuries**	**Rate/1000 Alberta Children**
Dislocations, sprains and strains	830–848	56754	24.76%	75.68
Superficial injury and contusions	910–924	54798	23.91%	73.01
Open wounds	870–897	43617	19.03%	58.16
Fractures	800–829	23302	10.17%	31.07
Intracranial injury	850–854	7033	3.07%	9.38
Burns	940–949	5838	2.55%	7.78
Foreign body	930–939	5568	2.43%	7.42
Poisoning	960–989	5110	2.23%	6.81
Crushing injury	925–929	3399	1.48%	4.53
Internal injury of chest, abdomen and pelvis	860–869	382	0.17%	0.51
Injury to nerves and spinal cord	950–957	248	0.11%	0.33
Injury to blood vessels	900–904	151	0.07%	0.20
Others	905–909 958–959 990–999	23024	10.04%	30.70

**Figure 2:**
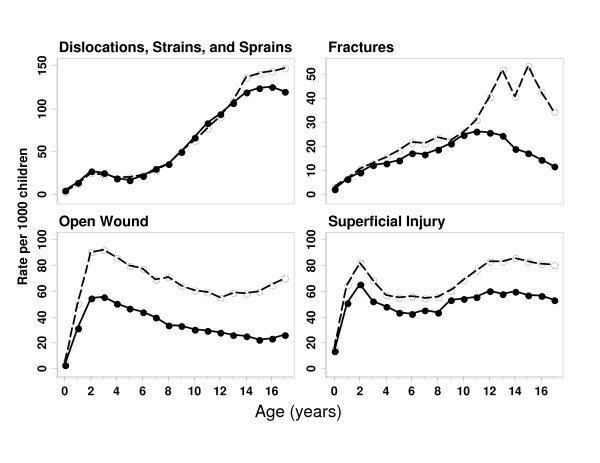
**Rates of Injury in Relation to Gender for the Top Four Types of Injury**. ○ indicates males, ● indicates females

Figure 2 illustrates the four most common types of injury in relation to gender, stratified by age, which were seen by a physician. It is clear that male/female patterns vary by type of injury.

#### Relationship between SES, gender, and types of childhood injuries

Table 4 shows that for the most part boys were significantly more likely than girls to experience all types of injury except for poisonings (odds ratio [OR] = 0.86) and dislocations, strains and sprains (OR = 1.0). Odds ratios describing the age and sex-adjusted relationship of SES with all types of injury show that, for the most part low SES children (i.e. those receiving a subsidy) had OR's significantly greater than 1, with maximum OR's of 1.60 for poisoning and 1.35 for burns, but a lower OR for dislocations, sprains and strains (0.89) and no difference for fractures (0.98).

**Table 4:** Relationships between gender, SES, and type of injury.

**Type of Injury**	**Adjusted OR^1 ^Males**	**95% CI**	**Adjusted OR**^2 ^**Low SES Group**	**95% CI**
Dislocations, sprains and strains	1.00	0.99–1.02	0.89	0.87–0.91
Superficial injury and contusions	1.26	1.24–1.28	1.11	1.09–1.14
Open wounds	1.86	1.83–1.90	1.14	1.11–1.16
Fractures	1.55	1.50–1.59	0.98	0.95–1.01
Intracranial injury	1.46	1.39–1.53	1.06	1.01–1.12
Burns	1.16	1.11–1.23	1.35	1.28–1.43
Foreign body	1.10	1.04–1.16	1.08	1.02–1.15
Poisoning	0.86	0.81–0.91	1.60	1.50–1.69
Crushing injury	1.13	1.05–1.21	1.22	1.13–1.31
Internal injury of chest, abdomen and pelvis	1.84	1.49–2.28	1.33	1.06–1.67

^1 ^Odds ratios (OR) reported here were adjusted for age and SES. Female = reference group.

^2 ^Odds ratios (OR) reported here were adjusted for age and gender. No subsidy = reference group.

The three sub-groups of healthcare premium subsidy were examined in relation to types of injury and for the most part children on welfare (social services) had OR's greater than other forms of subsidy (Table 5). Children with Treaty status had higher OR's than those from families receiving partial or total subsidy.

**Table 5:** Relationships between types of healthcare subsidy and types of injury.

**Type of Injury**	**Adjusted OR^1 ^** **Partial or Total Subsidy**	**95% CI**	**Adjusted OR^1 ^** **Treaty Status**	**95% CI**	**Adjusted OR**^1 ^ **Social Services**	**95% CI**
Dislocations, sprains and strains	0.84	0.81–0.86	0.90	0.87–0.94	1.04	1.00–1.08
Superficial injury and contusions	1.00	0.97–1.02	1.20	1.15–1.24	1.35	1.30–1.41
Open wounds	0.99	0.96–1.02	1.30	1.25–1.35	1.34	1.29–1.40
Fractures	0.89	0.85–0.93	1.16	1.10–1.23	0.99	0.92–1.05
Intracranial injury	1.01	0.94–1.08	1.06	0.95–1.17	1.22	1.10–1.35
Burns	1.16	1.08–1.25	1.49	1.36–1.65	1.69	1.53–1.87
Foreign body	1.04	0.96–1.13	1.04	0.93–1.17	1.24	1.11–1.39
Poisoning	1.22	1.13–1.33	2.04	1.86–2.24	2.09	1.89–2.31
Crushing injury	1.12	1.01–1.24	1.12	0.97–1.29	1.60	1.40–1.83
Internal injury of chest, abdomen and pelvis	1.08	0.79–1.47	1.23	0.81–1.89	2.15	1.50–3.07

^1 ^Odds ratios (OR) reported here were adjusted for age and gender. No subsidy = reference group.

#### Relationship between urban/rural residence and childhood injury

Odds ratios describing the relationship between SES, urban/rural residence, and overall injury rates show that there was a relationship between SES and injury for children living in urban Alberta when adjusted for age and gender (Table 6). When the three sub-groups of healthcare subsidy were examined it was evident that children whose families were on welfare or of Treaty status had considerably higher injury rates regardless of urban/rural residence, while those receiving partial/total subsidies and living in a rural setting had a significantly lower OR (OR = 0.85) than all other children.

**Table 6:** Relationships between urban/rural residence, types of healthcare subsidy, and all childhood injuries.

Residence	Adjusted OR1	95% CI
Urban		
Any Subsidy	1.09	1.07–1.11
Partial/Total Subsidy	1.01	0.99–1.03
Treaty Status	1.15	1.10–1.19
Social Services	1.23	1.20–1.27
Rural		
Any Subsidy	1.02	0.99–1.04
Partial/Total Subsidy	0.85	0.82–0.88
Treaty Status	1.21	1.18–1.25
Social Services	1.22	1.15–1.30

^1 ^Odds ratios (OR) reported here were adjusted for age and gender. No subsidy=reference group.

## Discussion

The AHW administrative database provided a unique opportunity to study childhood injury in Alberta since it includes virtually all children in the province and all injuries treated by a physician regardless of the setting. In addition, every child had potentially the same access to the universal healthcare system.

The findings indicate only slightly higher overall rates of child injury between children ages 0–17 years from low SES and higher SES families, but the rates were significantly higher for children of low SES from ages 0 to 9 years. When the three healthcare premium sub-groups were examined an interesting relationship appeared. Children whose family was receiving social services or who were of Treaty status were much more likely to have injuries treated by a physician at all ages than those receiving partial or total healthcare premium subsidy and the latter group actually had similar or lower injury rates when compared to children receiving no premium subsidy.

Further examination of the database provided more insight into the relationship between various types of childhood injury and SES. Children whose families had partial or total subsidised healthcare premiums have a disproportionately increased incidence of all types of injury except for dislocations, sprains and strains, and fractures. When the healthcare premium sub-groups were examined children whose families were on welfare had higher OR's for all types of injury than those receiving partial or total subsidy and most types of injury were also more frequent in those of Treaty status. The differences were especially evident with burns, poisonings, and for those on welfare, internal injuries. Thus, for what might be equivalent low incomes, children on social services or having Treaty status had higher injury rates for most types of injury.

Speculation about the mechanisms underlying these findings could include differences in environments, such as, less safe housing and neighbourhoods, and perhaps reduced use of safety measures at home and in play [18]. Children from lower socio economic backgrounds tend to live in higher population density neighbourhoods with more traffic and fewer playgrounds. These risks are intensified by the presence of social conditions associated with poverty: single parenthood, teenage parents, lower levels of parental education, large family size, lack of affordable day-care, and drug and alcohol abuse. These factors may add to the stresses of parenting and reduce the knowledge and experience needed to provide a safe environment for the child. Therefore, it is not surprising that children from lower SES families are more at risk for childhood injury.

A possible explanation for the lower rate of dislocations, strains, and sprains, and fractures among children of lower SES is that children of a higher SES may participate in more organised sports, and/or ride on snowmobiles, all terrain vehicles and cars, thereby leading to this type of injury. Interestingly, Lyons et al in Wales looked at fractures in children and concluded that although the rates were similar in both affluent and deprived areas, the causes were different with the more affluent areas having higher rates of sports related fractures and the poorer areas having more assault related injuries [9]. We can only say that in our study fractures were not significantly associated with SES. An examination of External Cause of Injury Codes (E-codes) would perhaps cast light on our observation, but E-codes were not recorded in this data set.

The findings from this present study appear to have uncovered an important reason for the variation in the literature on the relationship between SES and childhood injury. Most studies examining SES and injury have found a relationship between poverty and injury [12,13,19-22]. The finding is not universal; others have found no evidence of a relationship. For example, Addor et al demonstrated that socio-economic factors did not influence the occurrence of injury [23], and Larson et al. also showed no increase in risk of injury from children of lower income [24]. Our study, examining the type of childhood injury along with the SES of the family and having virtually the entire population of children of the Province of Alberta, presents a clearer picture of the relationship. Children whose parents receive partial or total healthcare subsidies appear to experience fewer injuries than those whose families are on welfare or who have Treaty status. Do the working poor hold different attitudes toward injury and childcare, or do they lack the means or time to take their child to a physician for care? Williamson and Fast reported that social assistance recipients seek medical treatment more frequently than the working poor [25] and these differences could contribute to the higher rates of childhood injury seen in this study.

Gender was a major factor to the pattern of childhood injury. After the age of one, males consistently presented for a physician consultation more frequently than females for all injuries. Information presented by Health Canada in the 1980's and 1990's indicated that more males were hospitalised and died from injuries than females in all provinces and territories [1], and Spady et al. also showed boys were more likely to be injured in Alberta [17]. Further examination of the types of injury and gender, with adjustment for age and SES, showed that males were more prone to all types of injury apart from dislocations, sprains and strains, and poisonings; the latter being more significantly more common in females and supported by Spady et al. [26]. Further research is required to determine whether males are more prone to injuries because of the different nature of childhood activities or differences in impulsiveness.

The types of injury occurring at differing ages during childhood often reflect various aspects of physical and mental development that influence susceptibility to injury. Several observations were made when rate of injuries in relation to age were examined. Two of the major categories of injury, superficial injury and contusions, and open wounds, demonstrated a dramatic rise in incidence about the age of one. During the infant and toddler period of growth there is a rapid increase in motor development; and there is a drive for autonomy and curiosity about the environment, thereby exposing the child to injury [27]. Superficial injury then decreased slightly during the four to eight year old age range before peaking during the teenage years. Often, school-age children seek social and peer acceptance and will engage in risk-taking behaviour. This coupled with an inadequate perception of speed, distance and strength may explain the increase in relatively minor injuries for this age range. The incidence of open wounds demonstrated a small increase in numbers but maintained an average rate of about 60–70/1000 children through the remaining years studied. Overall, the rates of childhood injury in Alberta during the fiscal year appear very high (e.g., about 300/1000 in the teenage years). Comparison with other studies and publications has not been possible as this study has captured all injuries treated by a physician and previous studies have only looked at hospitalisations.

When these data were examined in relation to the child's domicile, the analysis showed that injuries were more frequent in urban Alberta and in urban children with lower SES. The latter finding is supported in part by a previous study performed in Manitoba during 1994–1997 which indicated that injury hospitalisation among children living in low income areas of Winnipeg was 2.5 times higher than in the higher income areas[10]. The present study showed no relationship between SES and injury if the child lived in a rural community in Alberta. However, when the three sub-groups of subsidy were examined separately, children from families receiving partial/total subsidies and living in a rural setting had lower injury rates than other children for reasons that are not clear. Further research is required to determine why this group of children presented less frequently to a physician for an injury.

Limitations to the use of retrospective data depend on what data were collected and how it can be utilised. Unfortunately, there was no means available to determine the mechanisms of the injury with these data. However, these constraints are outnumbered by the advantages of utilising data that includes the individual economic status (healthcare premium payments); utilisation of diagnostic codes (number of times the healthcare system was accessed for injuries); and domicile of the child (rural/urban residence). Therefore, ecological fallacies were negligible in this study due to the individualisation of the data. This study was not able to differentiate between the "very poor", the "near poor" (families that do not qualify for healthcare premium subsidies) and those families with adequate incomes. However, there were distinct differences between children receiving welfare and the non-welfare poor (premium subsidy). It was recognised that Treaty status is not necessarily an indicator of poverty; the federal government pays the healthcare premium regardless of the person's income. First Nation people with Treaty status may be wealthy but often live in an environment where poverty is common. This study only counted one type of injury per episode; clearly, some of the injuries could be multiple. The data used for this study reflects only on children who were treated by a physician for an injury and obviously some injuries were treated at home. Children from lower SES circumstances, who sustain a minor injury, may be less likely to be brought in for physician treatment than their higher SES counterparts. Also, families in rural areas may be less likely to travel long distances to obtain treatment for a suspected minor injury than those of children living in urban domiciles. Therefore we are probably underestimating the true rates of injury. However, the opportunity to examine an individual level indicator of SES, with the possibility of moderate inaccuracies, outweighs the reduced reliability of aggregate data usage. Finally, healthcare services in Canada are universally accessible and because AHW is responsible for reimbursing physicians for consultations, the physician billing information was a reliable data set.

## Conclusion

The links between poverty and childhood injury are complex. The examination of this administrative database has highlighted some important patterns of childhood injury. Most types of injury were more frequent in children of lower SES. Analysis of the three types of the healthcare premium subsidy allowed a more comprehensive picture of childhood injury with children whose families are on welfare and those of Treaty status presenting more frequently for an injury-related physician's consultation than other children. Age and gender were also major influences on the rates of childhood injury. Injuries were more frequent in urban Alberta and in urban children of lower SES. Injuries cause much pain and suffering for children as well as their families and this is especially true in areas of socio-economic deprivation.

## List of Abbreviations

AHCIP Alberta Health Care Insurance Plan

AHW Alberta Health and Wellness

CI Confidence Interval

ICD-9 International Classification of Diseases, 9^th^. Revision

OR Odds Ratio

SES Socio-Economic Status

## Competing Interests

The author(s) declare that they have no competing interests.

## Authors' Contributions

**SJG **was the main author. She did most of the work as part of a thesis project for a Master of Science in Public Health Sciences

**DWS **assisted by providing access to the data, helping to edit the manuscript and some of the statistical analysis.

**DW **assisted by helping to edit the manuscript and providing important references for this report.

**TCW **supervised Susan Gilbride during her thesis and assisted with the writing and editing of the manuscript.

**LWS **provided the original data set to Donald Spady and read and approved the final draft of the article.

All authors read and approved the final manuscript.

## Pre-publication history

The pre-publication history for this paper can be accessed here:



## References

[B1] Health Canada (1997). For the Safety of Canadian Children and Youth: From Injury Data to Preventative Measures.

[B2] Health Canada (1999). Toward a Healthy Future: Second Report on the Health of Canadians.

[B3] UNICEF (2001). A league table of child deaths by injury in rich nations.

[B4] Scholer SJ, Hickson GB, Ray WA (1999). Sociodemographic factors identify US infants at high risk of injury mortality. Pediatrics.

[B5] Roberts I (1997). Cause specific social class mortality differentials for child injury and poisoning in England and Wales. Journal of Epidemiological and Community Health.

[B6] Carey V, Vimpani G, Taylor R (1992). Childhood injury mortality in New South Wales: geographical and socio-economic variations. Journal of Paediatric Child Health.

[B7] Dougherty G, Pless IB, Wilkins R (1996). Social class and the occurrence of traffic injuries and deaths in urban children. Canadian Journal of Public Health.

[B8] KIDS SAFE Connection (1999). Pediatric Major Trauma in Alberta 1995/96–1998/99.

[B9] Lyons RA, Dalahunty AM, Heaven M, McCabe M, Allen H, Nash P (2000). Incidence of childhood fractures in affluent and deprived areas: population based study. BMJ.

[B10] Brownell M, Friesen D, Mayer T (2002). Childhood injury rates in Manitoba. Canadian Journal of Public Health.

[B11] ICD.9.CM (1992). International Classification of Diseases, 9th Revision, Clinical Modification.

[B12] Faelker T, Pickett W, Brison RJ (2000). Socioeconomic differences in childhood injury: a population based epidemiologic study in Ontario, Canada. Injury Prevention.

[B13] Durkin MS, Davidson LL, Kuhn L, O'Connor P, Barlow B (1994). Low-income neighborhoods and the risk of severe pediatric injury: a small-area analysis in northern Manhattan. American Journal of Public Health.

[B14] Finkelstein MM (2004). Ecologic proxies for household income: How well do they work for the analysis of health and health care utilization?. Canadian Journal of Public Health.

[B15] Svenson LW, Woodhead SE, Platt GH (1993). Estimating the prevalence of asthma in Alberta : a study using provincial health care records. Chronic Diseases in Canada.

[B16] Alberta Health and Wellness (2000). Most recent version available.

[B17] Spady DW, Saunders DL, Schopflocher DP, Svenson LW (2004). Patterns of injury in children: a population-based approach. Pediatrics.

[B18] Baker SP, O'Neill B, Karpf R (1984). The Injury Fact Book.

[B19] Laing GJ, Logan S (1999). Patterns of unintentional injury in childhood and their relation to socio-economic factors. Public Health.

[B20] Durkin MS, Olsen S, Barlow B, Virella A, Connolly ES (1998). The epidemiology of urban pediatric neurological trauma, evaluation of, and implications for, injury prevention programs. Neurosurgery.

[B21] Gofin R, Lison M, Morag C (1993). Injuries in primary care practices. Archives of Diseases of Childhood.

[B22] Jolly DL, Moller JN, Volkmer RE (1993). The socio-economic context of child injury in Australia. Journal of Paediatric Child Health.

[B23] Addor V, Santos-Eggimann B (1996). Population-based incidence of injuries among preschoolers. European Journal of Pediatrics.

[B24] Larson CP, Pless IB (1988). Risk factors for injury in a 3-year-old birth cohort. American Journal of Diseases of Children.

[B25] Williamson DL, Fast JE (1998). Poverty status, health behaviours, and health: Implications for social assistance and health care policy. Canadian Public Policy.

[B26] Spady DW, Schopflocher DP, Svenson LW, Thompson AH (2005). Medical and psychiatric comorbidity and health care use among children 6 to 17 years old. Archives of Pediatric Adolescent Medicine.

[B27] National Centre for Injury Prevention and Control (2001). Injury Fact Book 2001–2002.

